# An entropy test for single-locus genetic association analysis

**DOI:** 10.1186/1471-2156-11-19

**Published:** 2010-03-23

**Authors:** Manuel Ruiz-Marín, Mariano Matilla-García, José Antonio García Cordoba, Juan Luis Susillo-González, Alejandro Romo-Astorga, Antonio González-Pérez, Agustín Ruiz, Javier Gayán

**Affiliations:** 1 Department of Quantitative Methods, Technical University of Cartagena, Paseo Alfonso XIII, 50, 30203, Cartagena, Spain; 2 Department of Structural Genomics, Neocodex, Avenida Charles Darwin, 6, 41092 Sevilla, Spain; 3 Department of Quantitative Economy I, UNED, Senda del Rey 11, 28040, Madrid, Spain

## Abstract

**Background:**

The etiology of complex diseases is due to the combination of genetic and environmental factors, usually many of them, and each with a small effect. The identification of these small-effect contributing factors is still a demanding task. Clearly, there is a need for more powerful tests of genetic association, and especially for the identification of rare effects

**Results:**

We introduce a new genetic association test based on symbolic dynamics and symbolic entropy. Using a freely available software, we have applied this entropy test, and a conventional test, to simulated and real datasets, to illustrate the method and estimate type I error and power. We have also compared this new entropy test to the Fisher exact test for assessment of association with low-frequency SNPs. The entropy test is generally more powerful than the conventional test, and can be significantly more powerful when the genotypic test is applied to low allele-frequency markers. We have also shown that both the Fisher and Entropy methods are optimal to test for association with low-frequency SNPs (MAF around 1-5%), and both are conservative for very rare SNPs (MAF<1%)

**Conclusions:**

We have developed a new, simple, consistent and powerful test to detect genetic association of biallelic/SNP markers in case-control data, by using symbolic dynamics and symbolic entropy as a measure of gene dependence. We also provide a standard asymptotic distribution of this test statistic. Given that the test is based on entropy measures, it avoids smoothed nonparametric estimation. The entropy test is generally as good or even more powerful than the conventional and Fisher tests. Furthermore, the entropy test is more computationally efficient than the Fisher's Exact test, especially for large number of markers. Therefore, this entropy-based test has the advantage of being optimal for most SNPs, regardless of their allele frequency (Minor Allele Frequency (MAF) between 1-50%). This property is quite beneficial, since many researchers tend to discard low allele-frequency SNPs from their analysis. Now they can apply the same statistical test of association to all SNPs in a single analysis., which can be especially helpful to detect rare effects.

## Background

The etiology of complex diseases is due to the combination of genetic and environmental factors, usually many of them, and each with a small effect. The identification of these small-effect contributing factors is still a demanding task, often requiring a large budget, thousands of individuals, and half-a-million or more genetic markers. Even so, success is not guaranteed. In the last decade, genetic association tests have become widely used, since they can detect small genetic effects. The current availability of genome-wide genotyping tools, combined with large collections of affected and unaffected individuals, has allowed for association analysis of the entire genome with the intention to detect even those small genetic effects (i.e., Odds-Ratios (OR) around 1.2) that influence common complex diseases.

We have seen recently a proliferation of genome-wide association (GWA) analyses, some of which are identifying even genes with only small or modest effect sizes ([1] for a review). Nonetheless, the genetic factors found so far do not explain the total heritability of these diseases. Perhaps, the genetic architecture of these diseases is more complex than previously thought, involving many more genes, each with a small effect, and interacting among them and with environmental factors in complex ways. There is also the possibility of a large background of rare mutations, each possibly having a relatively large effect, but at a very low frequency [2]. Clearly, there is a need for more powerful tests of genetic association, and especially for the identification of rare effects. This need will probably be exacerbated when low-cost whole genome sequencing becomes available, uncovering a large amount of rare variants in humans [3].

Although Information Theory was originally applied in the context of communication and engineering problems [4], entropy-based approaches have been also successfully applied to gene mapping. Specifically, there are information-theory-based tests implemented for population-based association studies using genotypic tests in case-control analysis and QTL analysis [5,6], gene-centric multimarker [7] and haplotype-based association studies [8] or epistasis analysis [9-12]. Moreover an entropy-based Transmission Disequilibrium Test (TDT) has also been described to conduct genome-wide studies in family trios [13].

In spite of these achievements, there is a scarce amount of simple and user-friendly computer programs to analyze and prioritize genome wide signals using entropy-based algorithms. Furthermore, a general entropy-based allelic test has not been described, studied and implemented in software to date. We have created a new genetic association test based on entropy that provides a general tool to conduct whole genome association studies. It is a new, simple, consistent and powerful test to detect genetic association of biallelic/SNP markers in case-control data, by using symbolic dynamics and symbolic entropy as a measure of gene dependence. Furthermore, we have implemented these algorithms in a software freely available to the scientific community. Using this computer program, named Gentropia, we have applied this entropy test, and a conventional test, to simulated and real datasets, to illustrate the method and estimate type I error and power of the test.

## Results

To illustrate the method we used data from the SNP Resource at the NINDS Human Genetics Resource Center DNA and Cell Line Repository http://ccr.coriell.org/ninds/. The original genotyping was performed in the laboratory of Drs. Singleton and Hardy (NIA, LNG), Bethesda, MD USA [14]. We have used data on 270 patients with Parkinson's disease and 271 normal control individuals who were genotyped for 396,591 SNPs in all 22 autosomal chromosomes using the Illumina Infinium I and Infinium II assays. Cases were all unrelated white individuals with idiopathic Parkinson's disease and age of onset between 55-84 years (except for 3 young-onset individuals). The control sample was composed of neurologically normal, unrelated, white individuals. To explore the properties of the entropy test, and compare it to an equivalent conventional chi-square test, we simulated and analyzed datasets with specific properties. To simulate the specific effect size of a genetic variant, we wrote an algorithm that fixes the odds-ratio (OR) attributed to the SNP, and either fixes or sets randomly the minor allele frequency (MAF) in controls. Subsequently it estimates the MAF in cases necessary to generate the desired OR. Then, specific genotypes are generated for cases and controls according to the estimated allele frequencies in each group, and assuming Hardy-Weinberg equilibrium. Most datasets include 500 cases and 500 controls, and SNP marker genotypes were simulated under different genetic models (OR equal to 1 (no effect), 1.25, 1.5 and 2), and different marker allele frequencies (MAF equal to 0.05, 0.2, and 0.4). Type I error of the statistical tests was evaluated in a dataset where 10,000 SNPs were simulated under the null hypothesis, with allele frequencies chosen randomly between 0 and 0.5, but each SNP had a similar MAF in the case and control groups. For the power analysis, each dataset contains 100 SNPs with specific OR and MAF. Finally, to evaluate low allele-frequency markers in more detail, we simulated datasets of 5000 cases and 5000 controls, and 1000 SNPs with a variety of effect sizes (OR equal to 1, 1.5 and 1.8) and allele frequencies (MAF = 0.01, 0.03 and 0.06).

The entropy allelic and genotypic tests can be compared to association tests used commonly in the field of Human Genetics. For a biallelic SNP marker, a test of association between the SNP and a disease can be computed by comparing the allelic or the genotypic frequencies in cases and controls. The conventional allelic test is a chi-square test statistic with 1 degree of freedom, while the conventional genotypic test is a chi-square test with 2 degrees of freedom.

### Null simulations

A simulated dataset, consisting of 500 cases and 500 controls, was analyzed with both conventional chi-square and entropy-based association tests. A total of 10,000 SNPs, with different allele frequencies (0 < MAF < 0.5), were simulated under the null hypothesis, that is, to have no effect on the trait. This analysis can reflect whether the new entropy-based association tests conform to the theoretical distribution. We counted the number of test-statistics that had values above the critical values of the expected distribution, to estimate the type I error for each test.

The conventional chi-square and the entropy-based tests, in both its genotypic and allelic versions, yield approximately the expected number of false positives (see Table 1), suggesting they all conform to the expected theoretical distributions (*χ*^2 ^with 1 or 2 degrees of freedom). The entropy-based test statistic was always equal or larger in value than the conventional chi-square test. On average, the entropy-based genotypic method increased the test-statistic in 0.047 chi-square units (a 1.7 percent), while the entropy allelic test exhibited an average increase of 0.003 chi-square units (a 0.1 percent).

**Table 1:** Type I Error for Conventional and Entropy tests.

Expected	Conventional Allelic	AL_i_	Conventional Genotypic	GE_i_
0.0500	0.0475	0.0478	0.0484	0.0514

0.0100	0.0109	0.0111	0.0099	0.0104

0.0010	0.0012	0.0013	0.0010	0.0010

AL = Entropy allelic test. GE = Entropy Gentotypic test.

### Power analysis

To estimate the power of both conventional and entropy-based tests, we carried out an analysis of simulated dataset of 500 cases and 500 controls. Sets of 100 SNPs were simulated under different alternative hypothesis, with different effect sizes (odds-ratios of 1.25, 1.5, and 2) and minor allele frequencies (0.05, 0.2 and 0.4). The entropy-based test statistic was always equal or larger in value than the conventional chi-square test, and therefore its power was also always equal or larger (see Tables 2 and 3). This increase in power is small, and it is more pronounced for the genotypic than for the allelic test. The gain in power with the genotypic entropy test tends to become apparent for larger chi-square values, or especially in markers with low allele frequency. For the allelic test, the entropy test is also sensibly more powerful for the *OR *= 2 and *MAF *= 0.05 simulation.

**Table 2:** Power (%) for conventional (CA) and entropy (AL) allelic tests for different Minor Allele Frequencies (MAF) and Odds-ratios (OR).

	MAF	0.05		0.2		0.4	
OR = 1.25	*α*	CA	AL_i_	CA	AL_i_	CA	AL_i_

	0.001	1	1	12	12	20	20

	10^-4^	0	0	2	3	2	3

	10^-5^	0	0	1	1	1	1

	10^-6^	0	0	1	1	1	1

	10^-7^	0	0	0	0	1	1

OR = 1.5							

	0.001	12	12	71	71	88	88

	10^-4^	6	6	40	40	72	72

	10^-5^	2	3	20	20	51	51

	10^-6^	0	0	10	10	30	30

	10^-7^	0	0	5	5	17	19

OR = 2							

	0.001	77	79	100	100	100	100

	10^-4^	48	49	100	100	100	100

	10^-5^	25	26	96	96	100	100

	10^-6^	19	19	90	91	100	100

	10^-7^	10	11	82	82	98	98

**Table 3:** Power (%) for conventional (CG) and entropy (GE) genotypic tests for different Minor Allele Frequencies (MAF) and Odds-ratios (OR).

	MAF	0.05		0.2		0.4	
OR = 1.25	*α*	CG	GE_i_	CG	GE_i_	CG	GE_i_

	0.001	0	1	8	9	10	10

	10^-4^	0	0	2	2	3	3

	10^-5^	0	0	2	2	1	1

	10^-6^	0	0	0	0	1	1

	10^-7^	0	0	0	0	0	0

OR = 1.5							

	0.001	8	9	51	51	81	81

	10^-4^	5	6	28	28	59	59

	10^-5^	0	2	11	11	35	37

	10^-6^	0	0	7	8	21	22

	10^-7^	0	0	3	4	7	9

OR = 2							

	0.001	61	67	100	100	100	100

	10^-4^	32	37	98	98	100	100

	10^-5^	20	21	93	93	100	100

	10^-6^	12	14	84	84	99	100

	10^-7^	4	8	63	66	97	97

Because the gain in power is correlated with the size of the chi-square statistic, we computed a "proportional power gain", that is, the difference between the entropy and the conventional chi-squares, divided by the conventional chi-square. This proportional gain allows us to compare the gain across the different simulated scenarios. As can be seen in Table 4, the average increase in power is only small, ranging between 0.1 and 9.7%, except for the genotypic test on low allele frequency SNPs, for which the power gain range is much larger (5.2-9.7%). In general, the gain in power increases when the OR increases and when MAF decreases.

**Table 4:** Allelic and genotypic entropy-tests gain power (%) for different Minor Allele Frequencies (MAF) and Odds-ratios (OR).

		Allelic Power	Genotypic Power
**OR**	**MAF**	**Gain (Mean%)**	**Gain (Mean%)**

1.25	0.4	0.1	0.3

1.5	0.4	0.2	0.8

2	0.4	0.6	2

1.25	0.2	0.1	0.8

1.5	0.2	0.4	1.2

2	0.2	1.1	2.2

1.25	0.05	0.3	9.7

1.5	0.05	0.7	5.3

2	0.05	1.7	5.2

Results show that the entropy test is similar or more powerful than the conventional chi-square test. The gain in power is small, and in some cases not different from the false-positive increase under the null hypotheses. Nonetheless, the entropy tests are an improvement over genotypic tests, for reasons discussed in the Discussion, and may become useful when power is limited, and especially, for the analysis of low allele-frequency SNPs.

### Low-allele frequency markers

To study in more detail the performance of the genotypic conventional and entropy tests in low-allele frequency markers, we simulated datasets of 5000 cases and 5000 controls, so there would be enough power to detect these rare effects. Each dataset included 1000 SNPs simulated under an specific effect size (*OR *equal to 1, 1.5 and 1.8) and allele frequency (MAF equal to 0.01, 0.03 and 0.06).

The analysis of the null-effect markers (OR = 1), reveals that both tests conform approximately well to the hypothetical null distribution for allele frequencies of 0.06 and 0.03. However, both tests are too conservative for very rare alleles, with minor allele frequencies around 1% (Table 5).

**Table 5:** Type I error for genotypic test with low minor allele frequencies (MAF).

	Expected	CG	*GE*_i_
MAF = 0.01			

	0.05	0.022	0.019

	0.01	0.000	0.000

MAF = 0.03			

	0.05	0.044	0.052

	0.01	0.008	0.013

MAF = 0.06			

	0.05	0.057	0.062

	0.01	0.013	0.013

CG = Conventional genotypic test. GE = Entropy genotypic test.

Table 6 confirms that both tests behave similarly when the study has enough statistical power, that is, for allele frequencies above 5%, and even for markers with lower frequency and large effect (MAF = 3% and OR = 1.8). In contrast, it is evident that the genotypic entropy test is more powerful than the genotypic conventional test for markers with rare (MAF = 0.01), or low allele-frequency (MAF = 0.03).

**Table 6:** Genotypic-Test Power (%) for different low minor allele frequencies (MAF) SNPs and Odds-ratios (OR).

	MAF	0.01		0.03		0.06	
	*α*	CG	GE_i_	CG	GE_i_	CG	GE_i_

OR = 1.5							

	0.05	65.6	81.4	100	100	100	100

	0.01	49.9	64.3	99.3	99.3	100	100

	10^-3^	24.3	33.5	95.1	95.3	100	100

	10^-4^	9.6	14.4	87.4	87.5	100	100

	10^-5^	3.0	5.2	74.6	75.1	100	100

	10^-6^	0.9	1.2	55.9	57.4	98.9	99.0

	10^-7^	0.3	0.4	39.5	40.9	95.8	95.8

OR = 1.8							

	0.05	87.4	99.1	100	100	100	100

	0.01	85.1	96.8	100	100	100	100

	10^-3^	77.2	88.6	100	100	100	100

	10^-4^	64.2	74.8	100	100	100	100

	10^-5^	44.7	55.4	100	100	100	100

	10^-6^	28	34.8	99.7	99.8	100	100

	10^-7^	14.4	20.5	98.9	98.9	100	100

CG = Conventional genotypic test. GE = Entropy genotypic test.

It is important to note here that the Fisher exact test is often used as a test of association for rare SNPs. For this reason, we have also compared the Fisher and Entropy tests, in their allelic and genotypic versions. For low frequency SNPs (MAF = 0.03) the results suggest that all four tests conform to their theoretical distribution (Tables 7 and 8). We find that Fisher and Entropy are quite similar for the allelic test, with the Entropy test being slightly more powerful (but also slightly more liberal) than Fisher. We conclude that both tests are essentially equivalent for the allelic test. However, both allelic tests are more powerful than any of the genotypic tests (Table 7).

**Table 7:** Fisher versus entropy allelic tests for different Odds-ratios (OR).

Allelic	MAF 0.03					
	**Type I error**		**Power**		**Power**	

	**OR = 1**		**OR = 1.5**		**OR = 1.8**	

**alpha**	**Fisher**	**Entropy**	**Fisher**	**Entropy**	**Fisher**	**Entropy**

0.05	0.052	0.057	100.0	100.0	100.0	100.0

0.01	0.011	0.013	99.8	99.8	100.0	100.0

1.E-03	0.001	0.001	97.7	98.1	100.0	100.0

1.E-04	0.000	0.000	91.4	92.2	100.0	100.0

1.E-05	0.000	0.000	81.5	83.0	100.0	100.0

1.E-06	0.000	0.000	66.3	67.3	99.9	99.9

1.E-07	0.000	0.000	49.3	50.5	99.5	99.6

MAF = Minor allele frequency.

**Table 8:** Fisher versus entropy genotypic tests tests for different Odds-ratios (OR).

Genotypic	MAF 0.03					
	**Type I error**		**Power**		**Power**	

	**OR = 1**		**OR = 1.5**		**OR = 1.8**	

**alpha**	**Fisher**	**Entropy**	**Fisher**	**Entropy**	**Fisher**	**Entropy**

0.05	0.046	0.051	100.0	100.0	100.0	100.0

0.01	0.010	0.013	99.4	99.3	100.0	100.0

1.E-03	0.001	0.000	95.8	95.3	100.0	100.0

1.E-04	0.000	0.000	88.5	87.5	100.0	100.0

1.E-05	0.000	0.000	77.7	75.1	100.0	100.0

1.E-06	0.000	0.000	61.1	57.4	99.8	99.8

1.E-07	0.000	0.000	45.1	40.9	99.2	98.9

MAF = Minor allele frequency.

Tables 8 and 9 describe the Genotypic test, for MAFs of 0.03 and 0.01. When comparing Fisher versus Entropy genotypic tests with low frequency SNPs (MAF = 0.03), power is also very similar, slightly better for Fisher than Entropy (Table 8).

**Table 9:** Fisher versus entropy genotypic tests tests for different Odds-ratios (OR).

Genotypic	MAF 0.01					
	**Type I error**		**Power**		**Power**	

	**OR = 1**		**OR = 1.5**		**OR = 1.8**	

**alpha**	**Fisher**	**Entropy**	**Fisher**	**Entropy**	**Fisher**	**Entropy**

0.05	0.036	0.022	86.0	81.4	99.9	99.1

0.01	0.030	0.000	71.7	64.3	98.0	96.8

1.E-03	0.000	0.000	41.8	33.5	91.9	88.6

1.E-04	0.000	0.000	20.1	14.4	80.1	74.9

1.E-05	0.000	0.000	8.5	5.2	64.0	55.5

1.E-06	0.000	0.000	2.3	1.2	43.8	34.8

1.E-07	0.000	0.000	0.7	0.4	25.1	20.5

MAF = Minor allele frequency.

Nonetheless, for very rare alleles (MAF<0.01), both tests are extremely conservative, more so the Entropy test, which consequently shows lower power for association than the Fisher test. In summary, it seems that both tests, Fisher and Entropy, are optimal to test for association with low-frequency SNPs (MAF around 1-5%), and both are conservative for very rare SNPs (MAF<1%).

These results alltogether suggest that symbolic entropy based tests are valid for testing for association, and do not create a significant bias under the null hypothesis. Moreover, the entropy tests are more stable than the conventional and Fisher exact tests regardless the allelic frequency. In addition, entropy tests are less expensive in computational terms than Fisher exact test.

### Parkinson disease

To illustrate the analysis method in a real dataset, we have analyzed a sample of 270 Parkinson disease patients and 271 controls, genotyped for 396,591 SNPs across the genome. This dataset includes SNPs with a wide variety of characteristics, such as different allelic and genotypic frequencies.

As we saw in the simulated datasets, the entropy tests are generally more powerful than the conventional tests. For these real data, we find some SNPs for which the entropy chi-square is lower than the conventional chi-square. However, these markers have low call rates in cases (lower than 45%), suggesting the presence of genotyping errors, and therefore would generally be excluded from association analysis. For the genotypic test, chi-square values (2 df) range between 0 and 41.95 for the conventional test, and 0-44.95 for the entropy test. On average, the entropy chi-square is 1.9% larger than the conventional test. If we consider the top-100 chi-square values for each test, there is 92% concordance in the SNPs that appear in these two rankings (irrespective of order within the ranking). The 8 SNPs chosen only by the conventional test still appear in the top 112 SNPs for the entropy test, revealing that the entropy test agrees well with the conventional test. Nonetheless, the 8 SNPs chosen only by the entropy test appear in ranks 105-359 in the conventional test. These SNPs far down the ranking of the conventional test have a common characteristic, they have a low frequency for the rare genotype (0-2 individuals only). As we saw for the null simulations, for low allele/genotype frequencies, the genotypic entropy test statistic is larger than the conventional chi-square, suggesting that the entropy test can help detect genetic effects in low allele/genotype frequency SNPs. For the allelic test, chi-square values (1 df) range between 0-30.35 for the conventional test, and 0-32.29 for the entropy test. Both tests agree well in chi-square size, with only a 0.1% difference on average. Both tests also agree on 96% of the SNPs in their top-100 ranking, and the 4 SNPs in disagreement, are ranked no lower than 106th in the other ranking. These tests are nearly identical, with the entropy test slightly more powerful.

## Discussion

Several entropy-based tests have been recently developed for population-based and family-based genetic association studies to perform gene mapping of complex diseases. However, to our best knowledge a simple and computationally feasible allelic entropy-based test useful for GWA studies is not available yet. Allelic and genotypic methods represent the gold-standard statistical test to start the prioritisation of markers during GWAS. The development of new and more powerful association tests can aid in the identification of small or rare effects, which may be widespread in the etiology of complex diseases, as shown in the recent GWAS [1]. To cover this need, we have developed a new likelihood ratio test of genetic association for biallelic markers such as SNP markers that is based on symbolic analysis and the relevant concept of entropy. Other authors, [8,5] and [6] among others, have used the concept of entropy for case/control association studies. In [8], the authors develop a statistic, namely *T*_*PE*_, that asymptotically follows a *χ*^2 ^distribution. In order to obtain the asymptotic distribution of *T*_*PE*_, they require entropy to be continuously differentiable with respect to the frequencies of the haplotypes, which represent a problem when the frequency of an haplotype is zero either in cases or in controls. In such a case then the haplotypes need to be grouped with others haplotypes which yield a decrease in statistical power. Moreover the *T*_*PE *_statistic also requires the estimation of an inverse matrix. Since this is not always possible, this inverse matrix has to be approximated by its generalized inverse, possibly introducing a bias in the statistic. Also, the computation of *T*_*PE *_is more expensive in computational running times than our entropy-based test *GE*_*i *_[6]. provides a measure for linkage disequilibrium (LD) between a marker and the trait locus, that is based on the comparison of the entropy and conditional entropy in a marker in extreme samples of population. Nevertheless, the authors do not give the distribution of the constructed measure, and hence it is not possible to assign a statistical significance to the procedure. Finally, [5], in the context of clusters of genetic markers, uses multidimensional scaling in conjunction with the Mutual Information (MI) between two discrete random variables. They use the fact that under the null of no association, MI can be approximated by means of a second order Taylor series expansion to a Gamma distribution. These entropy-based methods provide a tool to test for allelic and genotypic association between a marker and a qualitative phenotype. In these papers, the empirical size and power of the tests has not been computed nor compared in power with conventional tests.

The entropy test has several advantages over conventional tests: (1) It has been proved that the test is consistent. This is a valuable property since the test will asymptotically reject any systematic deviations between the distributions of cases and controls. (2) Importantly, the test does not require prior knowledge of parameters, and therefore can not be biased by potential decisions of the user. These properties, together with the fact that the test is simple, intuitive and fast in computational terms, make this test a theoretically appealing and powerful technique to deal with the detection of genetic association.

We have shown, both in simulated and real data, that the entropy and conventional tests, both in their genotypic and allelic versions, fit well their expected null distributions, or are even conservative for the detection of rare alleles (MAF ≤ 0.01). More so, the entropy genotypic test is more powerful than the conventional test, especially for those low-frequency SNPs. This is an important property, because there is a current need for tools to detect rare genetic effects.

The Fisher exact test is often used as a test of association for rare SNPs, although it is hard to program because of the complexity of its formula, and it is also computationally intensive. To make sure that the Entropy test is efficient also for rare SNPs, we have compared the Fisher and Entropy tests, in their allelic and genotypic versions. We have shown that the Entropy test is as powerful as the Fisher exact test for the analysis of low frequency SNPs (MAF between 1-5%). Therefore, this entropy-based test has the advantage of being optimal for most SNPs, only losing power respect to the Fisher test for very rare alleles (MAF<1%). This property is quite beneficial, since many researchers tend to discard low allele-frequency SNPs from their analysis. Now they can apply the same statistical test of association to all SNPs in a single analysis.

These entropy tests are easy to compute with the formulas provided in this paper, which can be incorporated into any genetic analysis tool. We are making freely available a simple software (Gentropia) to carry out these entropy-based genetic analyses. A linux version of the software can be downloaded from the following Website: http://www.neocodex.com/en/Gentropia.zip. The analysis is quite fast. For example, an association analysis of 1,000 SNPs on 10,000 individuals takes only 4 seconds on a 2.4 Ghz CPU; A genome-wide association analysis of 400,000 SNPs on 550 individuals takes 84 seconds, which is quite satisfactory

## Conclusions

In summary, this is an application of symbolic analysis and entropy to carry out a genome-wide association analysis. We have implemented this simple and fast method in a freely available software http://www.neocodex.com/en/Gentropia.zip. This entropy-based method to detect genetic association is more powerful than conventional tests, and can be especially useful in the detection of rare effects due to low-frequency genotypes. The method can be improved to include other tests of association (dominance, recessive, etc.), and covariates. Moreover, the method can be extended for the detection of epistasis.

## Methods

### Entropy Model

First we give some definitions and introduce the basic notation.

Let *P *be the population to be studied. Denote by *C *the set of cases with a particular disease in *P *and by *C*^*c *^the complementary, that is, the set of controls. Let *N*_*ca *_and *N*_*co *_be the cardinality of the sets *C *and *C*^*c *^respectively and let *N *= *N*_*ca *_+ *N*_*co *_be the total amount of individuals in the population. Each *SNP*_*i *_in each individual *e *∈ *P *can take only one of the three possible values, *AA*_*i*_, *Aa*_*i *_or *aa*_*i*_. Let *S*_*i *_= {*AA*_*i*_, *Aa*_*i*_, *aa*_*i*_}. Moreover, each individual *e *∈ *P *belongs to either *C *or *C*^*c*^, therefore we can say that a *SNP*_*i *_takes the value (*X*_*i*_, *ca*) if *e *∈ *C *or (*X*_*i*_, *co*) if *e *∈ *C*^*c*^, for *X*_*i *_∈ *S*_*i*_. We will call an element in *S*_*i *_× {*ca,co*} a *symbol*. Therefore we can define the following map

defined by *f*_*i*_(*e*) = (*X*_*i*_, *t*) for *X*_*i *_∈ *S*_*i *_and *t *∈ {*ca,co*}, that is, the map *f*_*i *_associates to each individual *e *∈ *P *the value of its *SNP*_*i *_and whether *e *is a control or a case. We will call *f*_*i *_a *symbolization map*. In this case we will say that individual *e *is of (*X*_*i*_, *t*) -type. In other words, each individual is labelled with its genotype, differentiating whether the individual is a control or a case.

Denote by(1)

and(2)

that is, the cardinality of the subsets of *P *formed by all the individuals of (*X*_*i*_, *ca*) -type and (*X*_*i*_, *co*) -type respectively. Therefore  is the number of individuals of *X*_*i*_-type.

Also, under the conditions above, one could easily compute the relative frequency of a symbol (*X*_*i*_, *t*) ∈ *S*_*i *_× {*ca*, *co*} by:(3)

and(4)

Hence the total frequency of a symbol *X*_*i *_is .

Now under this setting we can define the *symbolic entropy *of a *SNP*_*i*_. This entropy is defined as the Shannon's entropy of the 3 distinct symbols as follows:(5)

Symbolic entropy, *h*(*S*_*i*_), is the information contained in comparing the 3 symbols (i.e., the 3 possible values of the genotype) in *S*_*i *_among all the individuals in *P*.

Similarly we have the symbolic entropy for cases, controls and case-control entropy by(6)

and(8)

respectively.

### Construction of the entropy test

In this section we construct a test to detect gene effects in the set *C *of cases with all the machinery defined in Section 1. In order to construct the test, which is the aim of this paper, we consider the following null hypothesis:(9)

that is,(10)

against any other alternative.

Now for a symbol (*X*_*i*_, *t*) ∈ *S*_*i *_× {*ca*, *co*} and an individual *e *∈ *P *we define the random variable  as follows:(11)

that is, we have that  = 1 if and only if *e *is of (*X*_*i*_, *t*) -type,  = 0 otherwise. Therefore, given that an individual *e *is a case, *t = ca*, (respectively e is a control *t = co*), the variable  indicates whether individual *e *has genotype *X*_*i *_(taking value 1) or not (taking value zero).

Then  is a Bernoulli variable with probability of "success" either  if *t *= *ca *or  if *t *= *co*, where "success" means that *e *is of (*X*_*i*_, *t*) -type. Then we are interested in to know how many *e*'s are of (*X*_*i*_, *t*) -type for all symbol (*X*_*i*_, *t*) ∈ *S*_*i *_× {*ca*, *co*}. In order to answer the question we construct the following variable(12)

The variable  can take the values {0,1,2,..., *N*}. Therefore, it follows that the variable  is the Binomial random variable(13)

Then the joint probability density function of the 6 variables(14)

is:(15)

where *a*_1 _+ *a*_2_+ *a*_3_+ *a*_4_+ *a*_5_+ *a*_6 _=*N*. Consequently the joint distribution of the 6 variables  is a multinomial distribution.

The likelihood function  of the distribution (15) is:(16)

where . Also, since

it follows that the logarithm of this likelihood function remains as

In order to obtain the maximum likelihood estimators  and  of  and  respectively for all *i *= 1,2,3,, we solve the following equations(17)

to get that(18)

Then, under the null *H*_0_, we have that  and thus,

Therefore the likelihood ratio statistic is (see for example [15]):(19)

and thus, under the null *H*_0 _we get that *λ*_*i*_(*Y*) remains as:(20)

On the other hand, *GE*_*i *_= -2ln(*λ*_*i*_(*Y*)) asymptotically follows a Chi-squared distribution with 2 degrees of freedom (see for instance [15]). Hence, we obtain that the estimator  of *GE*_*i *_is:(21)

Therefore we have proved the following theorem.

**Theorem 1**. Let *SNP*_*i *_be a single nucleotide polymorphism. For a particular disease denote by *N *the number of individuals in the population, *N*_*ca *_the number of cases and by *N*_*co *_the number of controls. Denote by *h*(*C,C*^*c*^) the case-control entropy and by *h*(*S*_*i*_), *h*(*S*_*i*_, *ca*) and *h*(*S*_*i*_, *co*) the symbolic entropy in the population, in cases and in controls respectively, as defined in (5, 6 and 7). If the *SNP*_*i *_distributes equally in cases than in controls, then(22)

is asymptotically  distributed.

Let *α *be a real number with 0 ≤ *α *≤ 1. Let  be such that(23)

Then the decision rule in the application of the *GE*_*i *_test at a 100(1-*α*)% confidence level is:(24)

Furthermore, an entropy allelic test can be developed in a similar manner. More concretely, let now define the set *A*_*i *_= {*A*_*i*_, *a*_*i*_} formed by the two possible alleles that form the *SNP*_*i*_.

Let(25)

Denote by  and  the total allele frequency. Then we can easily define the allele entropies of a *SNP*_*i *_by(27)

Now, with this notation and following all the steps of the proof of Theorem 1, we get the following result.

**Theorem 2**. Let *A*_*i *_= {*A*_*i*_, *a*_*i*_} be the alleles forming a single nucleotide polymorphism *SNP*_*i *_. For a particular disease denote by *N *the number of individuals in the population, *N*_*ca *_the number of cases and by *N*_*co *_the number of controls. Denote by *h*(*C,C*^*c*^) the case-control entropy and by *h*(*A*_*i*_), *h*(*A*_*i*_, *ca*) and *h*(*A*_*i*_, *co*) the allele entropy in the population, in cases and in controls respectively. If the allele *A*_*i *_distributes equally in cases than in controls, then(28)

is asymptotically  distributed.

### Consistency of the entropy test

Next we prove that the *GE*_i _test is consistent for a wide variety of alternatives to the null. This is a valuable property since the test will reject asymptotically that the *SNP*_i _distributes equally between cases and controls whenever this assumption is not true. The proof of the following theorem can be found in Appendix section. Since the proof is similar for both statistics we only prove it for *GE*_i_.

**Theorem 3**. Let *SNP*_i _be a single nucleotide polymorphism. If the *SNP*_i _does not distribute equally in cases than in controls, then

for all real number 0 <*C *< ∞.

Since Theorem 3 implies *GE*_i _→ +∞ with probability approaching 1 always *SNP*_i _does not distribute equally in cases than in controls, then upper-tailed critical values are appropriated.

## Authors' contributions

MRM, MMG, and JAG conceived and designed the novel statistical test. MRM, AR, AGP and JG developed the analysis tool. JLSG and ARA implemented the software. MRM, AGP, AR and JG acquired and generated the datasets, analyzed the data and interpreted the results. MRM, AGP and JG wrote the paper. All authors read and approved the final manuscript

## Appendix: Proof of consistency

Proof of Theorem 3 First notice that the estimators ,  and , of *h*(*S*_*i*_, *ca*), *h*(*S*_*i*_, *co*) and *h*(*S*_*i*_) respectively, are consistent because ,  and  Denote by *H*_*i *_= *h*(*C*, *C*^*c*^) + *h*(*S*_*i*_) - *h*(*S*_*i*_, *ca*) - *h*(*S*_*i*_, *co*) and notice that *H*_*i *_can be written as

Hence, since -ln(*x*) > 1 - *x *for all *x *≠ 1 we get that

always(29)

for some *X*_*i *_∈ *S*_*i*_.

On the other hand, *H*_0 _is equivalent to(30)

Therefore under the alternative, *H*_1_, condition (29) is always satisfied and hence *H*_*i *_> 0.

Let 0 <*C *< ∞ be a real number and take *N *large enough such that(31)

Then it follows that(32)

Therefore, by (31) and (32) we get that(33)

as desired.
